# World's largest vaccination drive in India: Challenges and recommendations

**DOI:** 10.1002/hsr2.355

**Published:** 2021-08-18

**Authors:** Gladson Vaghela, Kapil Narain, Mashkur Abdulhamid Isa, Vaishnavi Kanisetti, Attaullah Ahmadi, Don Eliseo Lucero‐Prisno

**Affiliations:** ^1^ GMERS Medical College Gandhinagar Gujarat India; ^2^ Nelson R. Mandela School of Medicine, University of KwaZulu‐Natal Durban South Africa; ^3^ School of Health and Related Research University of Sheffield Sheffield UK; ^4^ Bhaskar Medical College Hyderabad India; ^5^ Medical Research Center, Kateb University Kabul Afghanistan; ^6^ Global Health Focus Asia Kabul Afghanistan; ^7^ Department of Global Health and Development London School of Hygiene and Tropical Medicine London UK; ^8^ Faculty of Management and Development Studies University of the Philippines (Open University) Los Baños Philippines

## 1 INTRODUCTION

The COVID‐19 pandemic has brought drastic changes in India, exposing the fragility of the already overburdened and under‐resourced health system. India has the third‐highest number of total COVID‐19 cases in the world (12.1 million), only surpassed by Brazil (12.6 million) and the United States (30.4 million) as of March 30, 2021.^1^ With a global death toll exceeding 2.8 million, countries worldwide are currently facing a second or third wave of the pandemic with lockdowns. Vaccines are a critical preventative measure in combating any public health crisis, especially for the current pandemic. Consequently, the Governments are approving their emergency use. As of March 30, 2020, Israel currently leads the race in vaccine rollout, administering 115.54 vaccine doses per 100 residents, leagues ahead of India, which has administered 4.57 vaccine doses per 100 residents.^1^ Vaccination in India, the second‐most populous country in the world (with 1.3 billion individuals)—is a colossal task with its unique share of complexity and challenges. This paper aims to explore the shortfalls of the COVID‐19 vaccination drive in India to develop potential solutions.

### 1.1 India's COVID‐19 vaccine drive strategy

On January 16, 2021, India launched the “World's largest Vaccination Drive” with two vaccine candidates: Covishield, the Indian version of the Oxford AstraZeneca vaccine, produced by the Serum Institute of India, and Covaxin, India's homegrown inactivated COVID‐19 vaccine, produced by the Pharma firm Bharat Biotech in collaboration with the Indian Council of Medical Research (ICMR) and the National Institute of Virology (NIV). India aims to immunize the following people in a phased manner: Health Care Workers (HCWs) and Front Line Workers (FLWs), followed by citizens more than 45 years of age and eventually citizens more than 18 years of age.^2^ This requires registration on a digital platform called COVID‐19 Vaccine Intelligence Network (Co‐WIN), after which information of the vaccination site to visit and time will be shared with the beneficiary. The number of individuals who receive vaccine doses is tracked on this system.^2^

## 2 GAPS AND CHALLENGES IN INDIA'S COVID‐19 VACCINE DRIVE

Globally, India has the potential to produce 60% of the vaccine stock.^3^ While the Government plans to inoculate around 300 million individuals of the priority group in the initial phase,^2^ the problems rarely revolve around production; instead, they center on equitable distribution. According to the World Bank, India's rural population of 895 million accounts for 65.5% of the total population.^4^ The ultimate goal should be to ensure that this vaccination drive is accessible to these rural populations, many of whom reside in remote villages and are often neglected. While COVID‐19 does not discriminate, society does.

### 2.1 Logistical and administrative requirements

India has instituted extensive Universal Immunization Programs (UIPs), notably in the field of polio.^2^ Yet, the lack of sufficient distribution of cold chains across different states of India poses the most significant challenge. Out of India's 29 000 cold chain points, 52% are concentrated in just six states, accounting for a third of the population: Maharashtra, Karnataka, Tamil Nadu, Rajasthan, Gujarat, and Andhra Pradesh.^5^ Furthermore, Maharashtra, Karnataka, Tamil Nadu, and Gujarat are among the top‐ranking states in terms of Gross State Domestic Product (GSDP),^6^ highlighting the disparity in the provision of infrastructure for this rollout. These four states account for 38% of the country's cold chain points but only 24% of the population. Around four cold chain points serve 100 000 population in Gujarat. In contrast, there is just one cold chain for the same amount of population in Jharkhand. Jharkhand, Uttar Pradesh, and Bihar are among the least served states in cold chain infrastructure.^5^

While there is a need for sufficient cold storage facilities, the lack of the required number of beneficiaries per session, lack of trained vaccinators, and low shelf life of the vaccine post vial opening has increased India's vaccine wastage. States like Jharkhand (37.3%), Chhattisgarh (30.2%), Tamil Nadu (15.5%), Jammu and Kashmir (10.8%), and Madhya Pradesh (10.7%) are reporting much higher vaccine wastage than the national average (6.3%), as of May 2021.^7^ India ranked within the 51 to 75 percentile range among 89 countries on effective vaccine management as per a global analysis by WHO‐UNICEF in 2018.^8^ It reports relatively low performance in following the required vaccine arrival procedures and using the Management Information System (MIS) to estimate vaccine demand. To successfully implement the COVID‐19 vaccine drive, the Government should pay immediate attention to these parameters.^8^

For efficient administration of the COVID‐19 vaccines in India, the authorities have incorporated CO‐WIN, a vaccine tracking and registration app to manage, deliver, and track the country's mass vaccination campaign. Eligible citizens can use either the CO‐WIN platform or Aarogya Setu, India's COVID‐19 contact tracing app, to book their vaccine appointment anytime, anywhere. Integration with Aarogya Setu enables CO‐WIN users to download a QR‐coded vaccine certificate and stay up‐to‐date about their COVID‐19 risk via contact tracing and self‐assessment features. But lack of proper internet penetration across rural communities will be a potent hurdle for the vaccination drive.^9^ A report by Nielson in 2019 concluded the overall internet penetration in India is at 36%, with almost 70% of the rural population being devoid of active internet facilities, with states like West Bengal, Bihar, Jharkhand, and Odisha having the lowest internet penetration.^10^

### 2.2 Vaccine hesitancy, misinformation & distrust

The spread of misinformation, particularly in India, is one of the biggest challenges for the COVID‐19 vaccination drive after cold storage and other logistic concerns. Globally, the rise in the misinformation during the COVID‐19 pandemic is evident, prompting WHO to convene the first infodemiology conference.^11^ The COVID‐19 pandemic has shown widespread misinformation than any other pandemic or epidemic in the past, especially with the rise in the content available on multimedia platforms.^12^, ^13^ After India's first reported case in January, major communication channels flooded with an infodemic of fake news regarding every facet of the pandemic, from its origin to its cure. In an analysis by Boom, a fact‐checking organization, out of 178 fact‐checks, 35% of them were fake videos and 29% of misleading images, exposing doctored information on a range of issues such as fake diagnosis and cure, falsified quotes by famous personalities, tailored guidelines, and promising home remedies among other.^14^

Misleading information specific to a particular religion is also rampant in India, where majority of the population practices Hinduism (more than 80% of the total population), followed by Islam (more than 13% of the total population).^15^ Members of Hindu community encountered fake news claiming meat of cow used for vaccine production. Likewise, members of the Muslim community encountered fake news claiming traces of pork in the vaccine.^16^ Fake communal rumors falsely alleging Muslims for spreading COVID‐19 virus were reported, following a mass gathering in Delhi in March 2020.^14^ Sensational stories by few journalists further aggravate this condition.^14^ Media in India, are often known to sensationalize stories.^17^ All these events promote fear within the community, intensifying the existing obstacles.

Due to the rapid development of COVID‐19 vaccines, hesitancy and distrust regarding safety and efficacy thrive. It is especially true for India's local vaccine, Covaxin, due to its emergency approval despite insufficient efficacy data.^18^ On January 3, 2021, India's top drug regulating body, the Central Drugs and Standards Committee (CDSCO), granted emergency approval for Covaxin^19^ even though phase III clinical trials were still ongoing and phase II studies were yet unpublished, then. It was later on published in *The Lancet* on March 3, 2021. The study stated that the results reported during the phase 2 study did not permit efficacy assessments and concluded that evaluating safety outcomes requires extensive phase 3 clinical trials.^20^, ^21^ This resulted in initial incidents of skepticism among HCWs, with less than 30% of New Delhi's HCWs across six government hospitals accepting vaccinations.^22^ In addition to this, since the onset of the pandemic, HCWs in India, especially doctors, have been the victims of abuse and violence.^23^, ^24^, ^25^ Loss of the beloved fosters a sense of dissatisfaction amongst family members and friends of patients resulting in anger and abuse on doctors.

### 2.3 Strengthening healthcare personnel infrastructure

Even if the challenges mentioned above are overcome, the vaccination drive is incomplete without qualified healthcare personnel. India has 2.9 million registered nursing personnel, translating to a ratio of 1.6 nurses per 1000 population, far below the WHO norms of 3 per 1000 population. Furthermore, the country is also deficient in doctors, at 0.9 per 1000 population (below the norm of 1 per 1000 population).^4^, ^26^ This is further compounded by the various challenges the HCWs have encountered during the pandemic, such as the shortage of personal protective equipment (PPE), long working hours, and even facing violence.^23^, ^24^, ^25^

While the Government has opted for a site‐based vaccination drive, the number of vaccinators and support staff needs to be ramped up sharply. The Indian Government plans to include Auxiliary Nurse Midwives (ANM) working on Universal Immunization Programmes for COVID‐19 vaccination purposes.^7^ In India, the public sector is the major provider of immunization activities, in which ANMs & Accredited Social Health Activist (ASHA) workers play key roles in delivering immunization services and by providing maternal and child health care in rural communities.^27^, ^28^ While the Government plans to rope in 154 000 ANMs, it still leaves a considerable gap for inoculating over a billion people.^7^ India has also deployed an army of 9,29 097 ASHA workers,^7^ for community‐based prevention, detection, and reporting of COVID‐19. However, with meager wages and an inadequate supply of PPEs, has left ASHA workers helpless.^29^

Due to the rapid production of the vaccine, healthcare personnel needs to be adequately equipped with knowledge and skills regarding vaccine rollout and protocols to report any side effects. The efficient use of online training platforms can be utilized to overcome such challenges. The Government of India has launched virtual training platforms like National Informatics Centre (NIC), Integrated Government Online training (iGOT) portal on digital infrastructure for knowledge sharing (DIKSHA) platform of Ministry of Human Resource Development (MHRD), and educational satellite (EDUSAT). These platforms contain training resources that HCWs can use to study and learn the required skills and review the materials to re‐strengthen their knowledge. It provides instructor‐led training modules, self‐learning training modules, and even a blended teaching approach.^9^

However, the lack of internet access in rural areas coupled with digital illiteracy could be a potent barrier to conducting online training. Therefore, state governments are directed to complete face‐to‐face training in situations where virtual training is not possible while also ensuring that the staff strictly adheres to COVID‐19 appropriate behavior for on‐ground training.^9^

## 3 POLICY RECOMMENDATIONS

The pandemic needs the support and coordinated efforts of both the public and the private sectors. While the Government pulls in the private sector in manufacturing vaccines,^2^ it should also effectively devise logistical needs and ensure continued transportation of vaccines with equitable distribution at its core.

At present, there are more than 30 000 Primary Healthcare Centers (PHCs) in India.^7^ Upgrading these centers with the required facility for vaccination can be beneficial to aid in the COVID‐19 vaccination process. As COVID‐19 strains our health system, PHCs can play a key role in preparedness and efficient vaccination service in rural areas. In Bhutan, within 2 weeks after launching its vaccination program, the authorities vaccinated 93% of adults.^30^, ^31^ Strong PHC networks across the country and partnerships with local government officials to reach citizens in even the most remote parts of the country aided this remarkable speed. Bhutan's Ministry of Health (MoH) used data from local governments and village leaders to determine the number of vaccines to distribute to each area, making COVID‐19 delivery easier.^30^ Bhutan's small population size is a major advantage over many other countries, like India; yet their strategies and experiences are a lesson for the world.

Public confidence and trust in COVID‐19 vaccines and those who administer them are just as important as the vaccines' safety and efficacy to ensure successful uptake. Effective PHC providers who have developed longitudinal, trusting relationships with their patients and communities have a key role to play in building demand for vaccines by transparently communicating both the advantages and possible side effects of vaccination, addressing misinformation around the vaccine, and tailoring messages to respect patients' beliefs and preferences.^32^

India should provide adequate cold storage in underserved areas and repurpose the existing facilities and resources to overcome the challenge. From repurposing ice‐cream freezers for storing and transporting vaccines in Canada^33^ to converting large venues like stadiums into super vaccination centers in the United States, countries worldwide are exploring novel approaches to meet the demand.^34^ India's Universal Immunization Program (UIP) currently consists of 85 634 equipment for vaccine storage at about 28 947 cold chain points across the country, which can be leveraged for COVID‐19 vaccination.^7^ But the Indian Government cannot forsake and divert all its resources for COVID‐19 vaccination, as other national immunization programs need to be run simultaneously. Hence, there is an urgent need to invest more in India's vaccine cold‐storage capacity. The Government could repurpose the existing food cold storage. There are currently 8186 cold storages with the capacity of 37.4 million Metrix Tonnes available in the country for storing perishable horticulture produce like fruits and vegetables.^7^

The Government can utilize social media as a powerful tool to curb the infodemic. This approach has been adopted in Finland, where health authorities collaborate with a network of social media influencers to help spread factual data about COVID‐19. Moreover, Finland claims to be the only country globally to have defined social media as “critical operators” during crisis—along with doctors, bus drivers, and other essential workers.^35^ The influencers' quick mobilization was possible because they have been part of Finland's emergency contingency plans for nearly 2 years. Social media influencers were added to the pool of essential actors a year and a half ago, after the media section of the national emergency supply organization realized traditional media would not be enough to reach the whole nation in a crisis.^36^ Embracing this mindset is critical, especially in this modern technological era. The Indian Government has incorporated celebrities and religious/faith‐based leaders in India's COVID‐19 Communication Strategies, including local folk songs, story‐telling, drama, and dance as the modes of community media. If successfully delivered, these will prove to be a critical move to get the right message in the community, especially in remote areas.^37^ This will help curb misinformation, resulting in more turnout of beneficiaries for vaccination, eventually causing less vaccine wastage.

As India grapples with the lack of healthcare personnel, especially doctors and nurses, it would require long term and systemic approach to address the root cause. Increasing availability of specialist HCWs in rural areas, especially at PHCs by utilizing communication technologies; monetary incentives for doctors providing rural services; ensuring holistic criteria for medical admission than merely merit‐based; and increasing post‐graduate seats in medical colleges could help overcome the problem^38^, ^39^ The authorities should recruit and invest in re‐strengthening allied health workers and CHWs, who have been an invaluable resource globally in previous pandemics and outbreaks, including the ongoing COVID‐19 pandemic, by providing them with necessary incentives, recognition and facilities.^29^

## 4 CONCLUSION

India has witnessed the successful eradication of smallpox and polio in the past.^2^ Such meticulous organization and administration can help India actualize the ambitious COVID‐19 vaccination drive. Authorities should invest in detailed planning for vaccinating, with a proper allocation of tasks and needs assessment, down to the lowest governance rung. Unless these measures are instituted, an appropriate estimate of the real challenges in COVID‐19 vaccine drive will not be understood, and the above challenges will remain unaddressed.

To enhance our knowledge and understanding and instill public trust and confidence, post‐marketing surveillance of the efficacy and safety of COVID‐19 vaccination is critical and requires long‐term monitoring.^40^ With the discovery of the delta variant in India,^41^ it is vital to enhance our understanding for better usage of the available interventions for treating COVID‐19. Owing to the ever‐changing scenario and advancement in the ongoing research, a robust data management infrastructure and knowledge sharing are vital across the nations. In addition, Governments worldwide need to harbor a sense of solidarity for equitable distribution of vaccines because no one is safe until everyone is safe.

## CONFLICT OF INTEREST

The authors have completed the ICMJE Unified Competing Interest form (available upon request from the corresponding author) and declare no conflicts of interest.

## AUTHOR CONTRIBUTIONS

Conceptualization: Gladson Vaghela, Attaullah Ahmadi, Don Eliseo Lucero‐Prisno III

Investigation: Gladson Vaghela, Vaishnavi Kanisetti, Kapil Narain

Project Administration: Gladson Vaghela

Supervision: Attaullah Ahmadi, Don Eliseo Lucero‐Prisno III

Writing‐Original Draft Preparation: Gladson Vaghela, Kapil Narain, Mashkur Abdulhamid Isa, Vaishnavi Kanisetti

Writing‐Review & Editing: Gladson Vaghela, Kapil Narain, Mashkur Abdulhamid Isa, Vaishnavi Kanisetti, Attaullah Ahmadi, Don Eliseo Lucero‐Prisno III

All authors have read and approved the final version of the manuscript.

## FUNDING

No funding from any institution or department.

## TRANSPARENCY STATEMENT

Gladson Vaghela affirms that this manuscript is an honest, accurate, and transparent account of the study being reported; that no important aspects of the study have been omitted; and that any discrepancies from the study as planned (and, if relevant, registered) have been explained.

## Data Availability

Data sharing does not apply to this article as no new data were created or analyzed in this study.
